# The Revised D‐A‐CH‐Reference Values for the Intake of Vitamin B_12_: Prevention of Deficiency and Beyond

**DOI:** 10.1002/mnfr.201801178

**Published:** 2019-01-28

**Authors:** Alexander Ströhle, Margrit Richter, Marcela González‐Gross, Monika Neuhäuser‐Berthold, Karl‐Heinz Wagner, Eva Leschik‐Bonnet, Sarah Egert, for the German Nutrition Society (DGE)

**Affiliations:** ^1^ Institute of Food Science and Human Nutrition Leibniz University Hannover Am Kleinen Felde 30 30167 Hannover Germany; ^2^ German Nutrition Society (DGE) Godesberger Allee 18 53175 Bonn Germany; ^3^ Institute of Nutritional Medicine University of Hohenheim Fruwirthstr. 12 70599 Stuttgart Germany; ^4^ ImFINE Research Group, Department of Health and Human Performance Universidad Politécnica de Madrid c/ Martín Fierro 7 28040 Madrid Spain; ^5^ Institute of Nutritional Science Justus‐Liebig‐University Goethestrasse 55 35390 Giessen Germany; ^6^ Department of Nutritional Sciences University of Vienna Althanstraße 14 1090 Vienna Austria

**Keywords:** cobalamine, holo‐transcobalamin, methylmalonic acid, reference value, vitamin B_12_

## Abstract

**Scope:**

The nutrition societies of Germany, Austria, and Switzerland are the joint editors of the “D‐A‐CH reference values for nutrient intake”, which are revised regularly.

**Methods and Results:**

By reviewing vitamin‐B_12_‐related biomarker studies, the reference values for vitamin B_12_ were revised in 2018. For adults, the estimated intake is based on the adequate serum concentrations of holotranscobalamin and methylmalonic acid. The estimated values for children and adolescents are extrapolated from the adult reference value by considering differences in body mass, an allometric exponent, and growth factors. For infants below 4 months of age, an estimated value is set based on the vitamin B_12_ intake via breast milk. The reference values for pregnant and lactating women consider the requirements for the fetus and for loss via breast milk. The estimated values for vitamin B_12_ intake for infants, children, and adolescents range from 0.5 to 4.0 µg d^−1^. For adults, the estimated values are set at 4.0 µg d^−1^, and for pregnant and lactating women, they are set at 4.5 and 5.5 µg d^−1^, respectively.

**Conclusion:**

Based on the data of several vitamin B_12_ status biomarkers studies, the reference value for vitamin B_12_ intake for adults is raised from 3.0 to 4.0 µg d^−1^.

## 1 Introduction

The D‐A‐CH ‘reference values for nutrient intake’^1^ are jointly issued by the nutrition societies of Germany, Austria, and Switzerland (the abbreviation D‐A‐CH arises from the initial letters of the common country identification for the countries Germany [D], Austria [A], and Switzerland [CH]). Reference value is a collective term for recommended intake (RI) values, estimated values, and guiding values. An RI value, according to its definition, meets the requirement of nearly any person (approximately 98%) of a defined group of metabolically healthy people. Estimated values are given when human requirements cannot be determined with desirable accuracy. Guiding values are stated in terms of aids for orientation.^1^

Reference values for nutrient intake are amounts that are assumed to

- protect nearly all healthy individuals in a population from deficiency‐related conditions
- ensure optimal physiological and psychological performance, and
- create a certain body reserve.^2^

Since 2012, the D‐A‐CH nutrition societies have published revised reference values for the intake of several nutrients^3^, ^4^, ^5^, ^6^, ^7^, ^8^, ^9^, ^10^, ^11^ but not yet for vitamin B_12_. The last update of the reference values for vitamin B_12_ given by the D‐A‐CH nutrition societies dates to a 2000 report.^12^ Since that time, a number of new scientific data have become available and have investigated the relationship between vitamin B_12_ intake and biomarkers of vitamin B_12_ status.^13^, ^14^, ^15^ From this background, there is a need to review and update the D‐A‐CH reference values. In 2018, the revised reference values for vitamin B_12_ intake were published in German. This paper provides a summary of this work.

## 2 Nutritional Physiology of Vitamin B_12_

Vitamin B_12_ is the collective term for a number of substances with qualitatively equivalent biological effects. These compounds consist of a corrin ring system that is similar to porphyrin with four reduced pyrrole rings and a central cobalt ion and are therefore referred to as cobalamins. Characteristically, all cobalamins have an α‐axial ligand at the cobalt ion consisting of a phosphoribosyl‐5,6‐dimethylbenzimidazole side chain. The β‐axial position can be occupied by various substituents. Depending on the group (R), a distinction is made between cyano‐ (R = CN), aquo‐ (R = H_2_O), hydroxo‐ (R = OH), methyl‐ (R = CH_3_), and adenosyl‐ (R = 5’‐deoxyadenosyl) cobalamin.^16^ Cobalamins are synthesized only by microorganisms, and humans and animals receive them solely through the food chain.^17^ In natural foods, vitamin B_12_ is present mainly as hydroxocobalamin and adenosylcobalamin and is found in milk as methylcobalamin.^18^ Due to its stability, cyanocobalamin is used in fortified foods as well as in pharmaceuticals and dietary supplements. Some pharmaceuticals and dietary supplements also contain hydroxocobalamin and methylcobalamin.^19^, ^20^

Humans metabolize cyanocobalamin and hydroxocobalamin into the two physiologically active coenzymes methylcobalamin and 5’‐deoxyadenosylcobalamin:^17^, ^19^

- **Methylcobalamin** is a coenzyme of cytosolic methionine synthase (EC 2.1.1.13) that catalyzes the remethylation of L‐homocysteine to L‐methionine. This reaction also involves folate as 5‐methyltetrahydrofolate (5‐MTHF), which is the actual methyl donor. Methylcobalamin that is bound to the methionine synthase is demethylated in this reaction and then again remethylated through 5‐MTHF. Accordingly, methylcobalamin is not only involved in the regulation of L‐homocysteine and L‐methionine but also required to provide free tetrahydrofolate (THF), which is an important coenzyme for the transfer of one‐carbon units, for example, in purine and pyrimidine synthesis.^21^
- **5’‐Deoxyadenosylcobalamin** is a coenzyme of the mitochondrial enzyme L‐methylmalonyl‐CoA mutase (EC 5.4.99.2). This enzyme isomerizes L‐methylmalonyl‐CoA to succinyl‐CoA, a central intermediate in the degradation of odd‐chain fatty acids and of the amino acids methionine, threonine, and isoleucine. In a first step, propionyl‐CoA is converted to D‐methylmalonyl‐CoA in a reaction catalyzed by the biotin‐dependent propionyl‐CoA carboxylase (EC 3.4.1.3). Thereafter, a racemase converts D‐methylmalonyl‐CoA to L‐methylmalonyl‐CoA.^22^ Furthermore, D‐Methylmalonyl‐CoA, which is a product of valine and thymine degradation, is also converted after epimerization to succinyl‐CoA through the L‐methylmalonyl‐CoA mutase reaction.

The largest vitamin B_12_ pool in the human body is located in the liver (approximately 50% of the total body pool). At a mean total body pool of 3 mg,^23^ this amount corresponds to a content of approximately 1.5 mg in the liver, with 61% present as adenosylcobalamin, 38% as hydroxycobalamin and only 1% as methylcobalamin.^18^ Significant quantities of vitamin B_12_ are also found in skeletal muscles (approximately 9%), bone marrow (approximately 4.5%) and bowel tissue (approximately 4.5%).^18^

In the blood, cobalamins are transported bound to the β‐globulins transcobalamin (TC)I (haptocorrin) or TC II. The most physiologically important carrier protein is TC II. The complex formed by vitamin B_12_ and TC II is referred to as holo‐TC II (simplified synonym holo‐TC).^24^ The half‐life of holo‐TC is 60 to 90 min^18^, ^25^; 10–30% of the circulating vitamin B_12_ is bound to TC II.^24^

## 3 Criteria to Assess the Vitamin B_12_ Status

There are different biomarkers for assessing vitamin B_12_ status. These biomarkers include the two status parameters vitamin B_12_ and holo‐TC in serum or plasma and the functional parameters methylmalonic acid (MMA) and homocysteine in serum.^21^, ^26^, ^27^, ^28^, ^29^, ^30^, ^31^, ^32^ However, none of these parameters are sufficient on their own to adequately assess vitamin B12 status.^29^ Furthermore, until now, no generally accepted cut‐off values exist for any of the biomarkers. Additionally, several groups have published different cut‐off values.^28^, ^33^, ^34^

### 3.1 Total Circulating Vitamin B_12_

A serum or plasma concentration of vitamin B_12_ between 148 pmol L^−1^ and 221 pmol L^−1^ indicates marginal supply,^28^ and a concentration of <148 pmol L^−1^ indicates vitamin B_12_ deficiency.^26^, ^28^, ^35^ A serum concentration of >221 pmol l^−1^ may be considered indicative of a sufficient vitamin B_12_ status in adults.^28^, ^36^ However, a serum concentration of >221 pmol L^−1^ may not necessarily mean that the vitamin B_12_ supply is sufficient since symptoms of vitamin B_12_ deficiency and/or a functional metabolic vitamin B_12_ deficiency may also be present.^21^, ^31^ Therefore, the total serum vitamin B_12_ concentration should be determined in combination with a functional parameter such as MMA.^29^, ^30^, ^37^

### 3.2 Circulating Holo‐TC

Circulating holo‐TC is responsible for supplying the tissues with vitamin B_12_.^38^ Thus, the serum holo‐TC concentration reflects the supply of metabolically active cobalamins.^32^, ^39^ A holo‐TC concentration between 40 and 100 pmol L^−1^,^36^ 40 and 200 pmol L^−1^ 39 or >37 and <190 pmol L^−1^ 40 is considered as the target concentration for a sufficient vitamin B_12_ status in adults. A holo‐TC concentration of >8.4 to <20 pmol L^−1^ is indicative of a possible vitamin B_12_ deficiency and a concentration of <8.4 pmol L^−1^ is indicative of a potential vitamin B_12_ deficiency.^40^ For a more reliable assessment of vitamin B_12_ supply, holo‐TC should be determined in combination with a functional parameter such as MMA.^29^, ^30^

### 3.3 Circulating MMA

Serum MMA concentration is considered as functional biomarker of vitamin B_12_ status. In the case of vitamin B_12_ deficiency, the serum concentration of MMA increases due to a reduced vitamin B_12_‐dependent isomerization of L‐methylmalonic acid to succinyl‐CoA through the enzyme L‐methylmalonyl‐CoA mutase.^29^, ^35^, ^41^ A MMA concentration of <210 28 or <270 nmol L^−1^ 36 is considered the target concentration for a sufficient vitamin B_12_ supply in adults. MMA concentrations of >350 to <840 nmol L^−1^ are indicative of an insufficient vitamin B_12_ status, and MMA concentrations of >840 nmol L^−1^ are indicative for a vitamin B_12_ deficiency.^40^ However, an increased MMA serum concentration may also occur due to impaired renal function. During pregnancy and at advanced age, MMA also increases independent of the vitamin B_12_ intake. Therefore, the specificity of MMA as a biomarker for vitamin B_12_ is limited.^29^, ^35^ Thus, MMA should only be used in combination with a status parameter such as holo‐TC.^29^, ^30^

### 3.4 Circulating Homocysteine

The remethylation of homocysteine to methionine is impaired if there is an insufficient vitamin B_12_ supply; consequently, plasma homocysteine concentration increases.^42^ However, the homocysteine concentration is influenced not only by vitamin B_12_ but also by folate, vitamin B_6_, and riboflavin (vitamin B_2_). Insufficient supply of these vitamins can also lead to increased homocysteine concentrations.^43^ The latter may also occur as a consequence of renal insufficiency, dehydration, and cystathionine β‐synthase deficiency as well as certain polymorphisms of the methylenetetrahydrofolate reductase.^44^ Therefore, the homocysteine concentration is a sensitive but non‐specific functional marker of vitamin B_12_ status.^26^ Accordingly, the homocysteine concentration is suitable to assess the vitamin B_12_ status only in combination with a vitamin B_12_ status parameter (serum holo‐TC or vitamin B_12_).^30^ In general, a plasma homocysteine concentration of <12 µmol L^−1^ in adults is considered to be adequate.^45^

## 4 Bioavailability of Vitamin B_12_

The bioavailability of vitamin B_12_ mainly depends on the intake level and decreases with an increasing dose.^46^ The approximate absorption rates for the intake of 1, 5, 10, and 20 µg are 50%, 20%, 10%, and only 5%, respectively.^47^ In addition to the dosage, the food matrix determines the absorption rate of vitamin B_12_.^17^, ^47^ The absorption rate when consuming lamb with a vitamin B_12_ content of 2.6 µg per 100 g is 56–89%, and for fish (rainbow trout with 4.9 µg per 100 g), the absorption rate is 42%. For cow's milk (0.4 µg per 100 g), a bioavailability of 65% has been determined.^17^ The absorption rate for eggs with an average vitamin B_12_ content of 1.3 µg per 100 g is <9%.^17^ Based on a meta‐regression analysis of eight bioavailability studies, absorption rates of 37% and 29% were determined for an intake of 1.2–3.1 µg vitamin B_12_, respectively, per meal complying with the usual dietary habits.^46^ At an average vitamin B_12_ intake of 1.2 µg per meal, the bioavailability is 37%,^46^ which means that for a mixed diet, which contains vitamin B_12_ also from foods with lower bioavailability,^17^ an availability of 35–40% may be assumed.

Vitamin B_12_ produced by colon bacteria in the human gut cannot be absorbed due to missing receptors.^48^

## 5 Derivation of the Reference Values for the Intake of Vitamin B_12_

### 5.1 Adults

#### 5.1.1 Adults under 65 Years of Age

As criteria to derive the reference value of vitamin B_12_ intake in adults, the serum concentrations of holo‐TC (≥ 40 pmol L^−1^) and of the functional parameter MMA in the desirable range of <270 nmol L^−1^ are considered as the most important ones. These criteria are based on the results of two studies, in which the adequate vitamin B_12_ intake was determined using the serum concentrations of total vitamin B_12_ and holo‐TC as well as the functional parameters MMA and homocysteine.^13^, ^14^ In the cross‐sectional study by Bor et al.^13^ with subjects aged 18 to 50 years, an average serum holo‐TC concentration of 65 pmol L^−1^ was reached with a daily median intake of 4.2 µg vitamin B_12_; higher intake levels were not associated with higher holo‐TC concentrations. The serum concentration of vitamin B_12_ reached an average of 325 pmol L^−1^. For the functional parameters MMA and homocysteine, average concentrations of 210 nmol L^−1^ and 8 µmol L^−1^, respectively, were measured,^13^ which indicate adequate vitamin B_12_ supply.^28^ The intake of more than 7 µg d^−1^ during this study did not result in a further increase in the plasma vitamin B_12_ concentration. With an intake of 7 µg d^−1^, the lowest serum concentrations of MMA and homocysteine were approximately 190 nmol L^−1^ and approximately 7 µmol L^−1^, respectively.^13^

More data on the dose‐response relationship between the vitamin B_12_ intake and the serum concentrations of MMA, homocysteine and vitamin B_12_ are available from a randomized controlled intervention study including 231 subjects.^14^ In this study, the lowest MMA and homocysteine concentrations were 190 nmol L^−1^ and 8.2 µmol L^−1^, respectively, with an intake of approximately 7 µg vitamin B_12_ d^−1^ (4 µg d^−1^ from foods plus 3.4 µg d^−1^ from a supplement). This dietary intake resulted in a serum concentration of vitamin B_12_ of approximately 330 pmol L^−1^. At a dietary intake of 4.0 µg vitamin B_12_, the serum concentrations of vitamin B_12_, MMA, and homocysteine were approximately 300 pmol L^−1^, 220 nmol L^−1^, and 8.3 µmol L^−1^, respectively.^14^

Based on the data from the studies by Bor et al.^13^ and Pentieva et al.^14^ as well as on the target concentrations for total serum vitamin B_12_ (>221 pmol L^−1^), holo‐TC (≥40 pmol L^−1^), MMA (<270 nmol L^−1^), and homocysteine (<12 µmol L^−1^), the estimated value for the vitamin B_12_ intake is 4.0 µg d^−1^ for men and women within the age range of 19 and 65 years (**Table** 1).

**Table 1:** Estimated values for adequate intake of vitamin B_12_

Group	Vitamin B_12_
**Infants**	µg d^−1^
0 to under 4 months	0.5
4 to under 12 months	1.4
**Children and adolescents**	
1 to under 4 years	1.5
4 to under 7 years	2.0
7 to under 10 years	2.5
10 to under 13 years	3.5
13 to under 15 years	4.0
15 to under 19 years	4.0
**Adults**	
19 to under 25 years	4.0
25 to under 51 years	4.0
51 to under 65 years	4.0
65 years and older	4.0
**Pregnant women**	4.5
**Lactating women**	5.5

#### 5.1.2 Adults above 65 Years of Age

In a study by Bor et al.^15^ on osteoporotic women and women at risk of osteoporosis at the age of 41 to 75 years, a median holo‐TC serum saturation of 119 pmol L^−1^ was achieved at a daily intake of 6 µg vitamin B_12_. At this dose, the median serum concentration of vitamin B_12_ was 380 pmol L^−1^. Median concentrations of MMA and homocysteine were 120 nmol L^−1^ and 9.75 µmol L^−1^, respectively,^15^ which indicate adequate vitamin B_12_ supply.^28^ However, 36% of the subjects had a gastric pH of ≥3 or other indications of gastric dysfunction.^15^ Those dysfunctions could cause an inhibition of gastric acid and pepsinogen secretion, which results in a reduced release of free vitamin B_12_ from food proteins, hence decreasing intestinal absorption of the cobalamin protein complexes from food. Furthermore, the reduced acid secretion leads to an alkalinization of the small intestine, which may result in bacterial overgrowth and thus to a further decrease of the bioavailability of vitamin B_12_.

A cross‐sectional study in German women at the age of 60 to 70 years (*n* = 178) showed that at an average intake of 5 µg d^−1^, 43% of the women had a serum cobalamin concentration of less than 258 pmol L^−1^.^49^ This concentration was defined in the study as a limit for sufficient supply. To derive the reference values for vitamin B_12_ intake, a concentration range of 148 to 221 pmol L^−1^ was defined as suboptimal, while a concentration of >221 pmol L^−1^ indicated sufficient supply. However, only 10% of the subjects in the study by Wolters et al.^49^ had an MMA concentration >271 nmol L^−1^. An MMA concentration of <270 nmol L^−1^ was considered the target concentration for a sufficient supply.

Currently, the available data are not sufficient to derive reference values for adults above 65 years of age for vitamin B_12_ intake that are different from those for younger adults. Thus, the estimated value for vitamin B_12_ intake for adults above 65 years of age does not differ from that for younger adults independent of gender and is set to 4.0 µg d^−1^ (Table 1).

### 5.2 Children and Adolescents

No data are available regarding the vitamin B_12_ requirement for children and adolescents. Therefore, the reference values for children and adolescents are based on the values compiled for adults and take into account the differences in body weight, an allometric exponent and the growth factors to consider the requirements for growth (**Table** 2). Growth factors at the different age groups were calculated as the proportional increase in protein requirement for growth relative to the maintenance requirement according to the WHO.^1^, ^50^ When using the age groups and reference body weights the D‐A‐CH reference values are based upon,^1^ the resulting estimated values for vitamin B_12_ intake range from 1.5 µg d^−1^ (for 1 to under 4 year olds) to 4.0 µg d^−1^ (for 15 to under 19 year olds) (Table 2).

**Table 2:** Estimated values for vitamin B_12_ intake for infants (4 to under 12 months), children and adolescents considering differences in body weight, allometric exponent, and growth factors

Age [years]	Gender	Reference body weight [kg]^a^, ^1^	Growth factor^b^, ^1^	Vitamin B_12_ intake considering reference body weight, allometric exponent and growths factor^c^ [µg d^‒1^]	Estimated value for vitamin B_12_ intake (rounded) [µg d^−1^]
4 to under 12 months	m	8.6	0.70	1.40	**1.4**
	f	7.9	0.70	1.49	
1 to under 4	m	13.9	0.25	1.47	**1.5**
	f	13.2	0.25	1.60	
4 to under 7	m	20.2	0.06	1.66	**2.0**
	f	20.1	0.06	1.87	
7 to under 10	m	29.3	0.13	2.34	**2.5**
	f	28.7	0.13	2.60	
10 to under 13	m	41.0	0.13	3.01	**3.5**
	f	42.1	0.11	3.41	
13 to under 15	m	55.5	0.10	3.66	**4.0**
	f	54.0	0.07	3.95	
15 to under 19	m	69.2	0.07	4.20	**4.0**
	f	59.5	0.02	4.07	

a The reference values for body weight correspond to the median body weight determined in the *German Health Interview and Examination Survey for Children and Adolescents in Germany* (KiGGS; 2003–2006).^126^ In each case, the values reflect the midpoint of the respective age range

b Growth factors at different ages were calculated as the proportional increase in protein requirement for growth relative to the maintenance requirement according to WHO^1^, ^50^

c Calculated from: estimated value_adults_ x (reference body weight_infants/children/adolescents_/reference body weight_adults_)^0.75^ x (1 + growth factor)

Estimated value_adults_: 4.0 µg d^−1^ (Table 1). Reference body weight_adults_ (age group 25 to under 51 years): men 70.7 kg, women 60.0 kg.^1^

Example: Estimated value_Girls, 1 to under 4 years_ = 4.0 µg d^−1^ × (13.2 kg per 60.0 kg)^0,75^ × (1 + 0.25) = 1.60.

### 5.3 Infants

The reference values for the intake of vitamin B_12_ for infants aged 0 to under 4 months were derived based on the vitamin B_12_ content of breast milk, which is considered to be the optimal diet for infants.^51^, ^52^ The cobalamin content of breast milk varies depending on the duration of breastfeeding^53^ and maternal vitamin B_12_ supply.^54^ It declines continuously during the lactation period.^55^ In a Danish study, in which the majority of the women had taken a multivitamin supplement (1.0–4.5 µg vitamin B_12_ d^−1^), the vitamin B_12_ content of the breast milk was 0.1 µg per 100 mL two weeks postpartum and 0.04 µg per 100 mL four months postpartum^53^ with a median vitamin B_12_ content in breast milk in the first four months of 0.07 µg per 100 mL.^53^ Assuming that the median vitamin B_12_ content of breast milk during the first four months is 0.07 µg per 100 mL, an exclusively breastfed infant receives 0.53 µg d^−1^ vitamin B_12_ from breast milk of mothers with adequate vitamin B_12_ supply at an average breast milk intake of 750 mL d^−1^.^56^ Therefore, the estimated value for the adequate intake of vitamin B_12_ for breastfed infants aged 0 to under 4 months is set at 0.5 µg d^−1^ (**Table** 3).

**Table 3:** Calculation of the estimated value for vitamin B_12_ for breastfed infants aged 0 to under 4 months

Age [month]	Vitamin B_12_ content of breast milk^a^ [µg per 100 mL]	Breast milk intake^b^ [mL d^−1^]	Vitamin B_12_ intake given a breast milk intake of 750 mL d^−1^ [mg d^−1^]	Estimated value for vitamin B_12_ intake (rounded) [mg d^−1^]
0 to under 4 months	0.07	750	0.53	**0.5**

a [53]

b [56].

The consumption of breast milk declines with the introduction of solid foods. Since no data are available from Germany with regard to vitamin B_12_ intake via solid foods, the estimated value for infants aged 4 to under 12 months is based on the values compiled for adults and considers differences in the body weight, an allometric exponent and a growth factor to consider the requirements for growth. An estimated value of 1.4 µg d^−1^ vitamin B_12_ for infants aged 4 to under 12 months was derived (Table 2).

### 5.4 Pregnancy

The requirement of pregnant women is slightly increased to meet the vitamin B_12_ requirement of the fetus. It was estimated that the fetus accumulates 0.1 to 0.2 µg vitamin B_12_ per day.^57^ Considering an absorption rate of 35–40%, the estimated value for vitamin B_12_ intake for pregnant women is therefore set to 4.5 µg d^−1^ (Table 1).

### 5.5 Lactation

In comparison to the vitamin B_12_ requirement of non‐lactating women, the requirement of lactating women is increased due to vitamin B_12_ secretion from breast milk. Approximately 0.5 µg d^−1^ vitamin B_12_ is secreted with breast milk (see 5.3 Infants). Therefore, considering an absorption rate of 35–40%, lactating women require an additional 1.5 µg d^−1^. Thus, the estimated value for vitamin B_12_ intake during lactation is 5.5 µg d^−1^ (Table 1).

## 6 Preventive Aspects

In the following paragraphs, currently available data on vitamin B_12_ are outlined in association with some health‐related aspects. Dietary reference values are aimed at healthy individuals; thus, the requirements of patients are not addressed.

### 6.1 Vitamin B_12_ and Cardiovascular Diseases

Since metabolic vitamin B_12_ deficiency impairs the remethylation of homocysteine to methionine, serum homocysteine concentration increases.^42^ It is well known that homocysteine is an independent risk factor for ischaemic heart disease and ischaemic stroke.^58^, ^59^, ^60^ By increasing the homocysteine concentration by 5 µmol L^−1^, the risk of coronary events increases by 18%.^60^ With an increase of 3 µmol L^−1^, the risk of stroke increases by 19%.^59^ However, it is not clear, whether this observation reflects a causal relationship.^61^ Nevertheless, genetic association studies,^62^, ^63^, ^64^ in vitro *s*tudies and animal studies indicate a causal link.^45^, ^65^ In contrast, the results from clinical intervention studies with vitamin B_12_, vitamin B_6_ and folate are contradictory. On the one hand, supplementation with B vitamins has the potential to reduce the relative risk of stroke by approximately 10%,^66^, ^67^ but vitamin B_12_ alone does not seem to exert a preventive effect.^67^ Additionally, vitamin B_12_ supplementation was not associated with a reduced risk of coronary events and cardiovascular diseases.^66^, ^68^

### 6.2 Vitamin B_12_ and Cancer

Vitamin B_12_ is essential for DNA methylation, genome integrity, and chromosomal stability, which suggests a preventive effect of vitamin B_12_ regarding the development of cancer.^69^ For breast cancer, a meta‐analysis of 18 observational studies showed an inverse association with the risk of disease and vitamin B_12_ intake but not for serum vitamin B_12_ concentration.^70^ Furthermore, no association was observed between dietary vitamin B_12_ intake^71^ or vitamin B_12_ status^72^ and the risk of endometrial cancer and colorectal carcinoma.^73^, ^74^, ^75^, ^76^, ^77^ Another meta‐analysis investigated the association between vitamin B_12_ serum concentration and the risk of renal cell cancer using data from seven cohorts. Compared with the group with the lowest intake, subjects with the highest intake had a relative renal cell cancer risk of 0.72 (95% CI  =  0.52–1.00).^78^

On the other hand, it is discussed that increased serum vitamin B_12_ concentration is associated with a higher risk of cancer. For example, in a meta‐analysis of observational studies, there was a positive association between vitamin B_12_ serum concentration and the risk of prostate cancer.^79^ In a cohort study, an increased serum vitamin B_12_ concentration (>600 pmol L^−1^) was associated with an elevated total risk of cancer incidence. The risk was highest within the first year of follow‐up.^80^ However, this association might be due to the increased intake of animal‐based foods, which is associated with high vitamin B_12_ intake (“spurious correlation”). Another cause for this association may be cancer types that are already present but not yet diagnosed, as some cancer types are associated with higher serum vitamin B_12 _concentrations.^81^

### 6.3 Vitamin B_12_, Miscarriages, and Neural Tube Defects

In observational studies, an insufficient supply of vitamin B_12_ during pregnancy is associated with an increased risk of miscarriage.^82^, ^83^ Furthermore, maternal vitamin B_12_ status is associated with the occurrence of neural tube defects in the child.^84^, ^85^ However, it remains unclear whether this observation reflects a causal relationship.^86^

### 6.4 Vitamin B_12_ and Bone‐Related Diseases

Vitamin B_12_ deficiency and hyperhomocysteinaemia are linked with the stimulation of osteoclast activity and accelerated degradation of the bone matrix.^87^, ^88^, ^89^, ^90^ The data from observational studies on the association between vitamin B_12_ status or vitamin B_12_ intake and bone mineral density, the risk of fracture or bone turnover markers are inconsistent.^88^ In a meta‐analysis of four prospective studies, for each increase of total serum vitamin B_12_ concentration by 50 pmol L^−1^, a 4% reduced fracture risk was calculated (95% CI  =  0.92–1.00).^91^ A meta‐analysis of four randomized controlled intervention studies indicates that the effect of vitamin B_12_ on the risk of fracture depends on the basal homocysteine concentration. A protective effect of vitamin B_12_ supplementation may be expected only at homocysteine concentrations >15 µmol L^−1^ (75% risk reduction; 95% CI  =  0.12–0.53). There is no evidence that vitamin B_12_ supplementation in excess of the individual requirement for subjects with adequate supply of folate and vitamin B_12_ at homocysteine concentrations <15 µmol L^−1^ results in a further fracture risk reduction.^92^

### 6.5 Vitamin B_12_, Neurodegenerative Diseases, and Cognitive Impairment

There is also a discussion about the question of whether functional vitamin B_12_ deficiency, which is characterized by decreased concentrations of holo‐TC and increased concentrations of MMA and/or homocysteine, is associated with an increased risk of neurodegenerative diseases and cognitive impairment.^93^, ^94^ Several epidemiological studies showed a positive correlation regarding MMA concentrations and an inverse association regarding holo‐TC concentrations with the development or progression of dementia, while there is only a low or no association between total vitamin B_12_ concentrations and disease risk.^72^, ^74^, ^75^, ^93^, ^94^ In older people, vitamin B_12_ deficiency is also associated with cerebral atrophy.^95^, ^96^ Vitamin B_12_ supplementation (0.5 mg d^−1^) in combination with folate (0.8 mg d^−1^) and vitamin B_6_ (20 mg d^−1^) in older people could slow cerebral atrophy over two years. This effect is, however, dependent on baseline homocysteine concentration.^97^

The concentrations of holo‐TC and MMA have proved to be good predictors of cognitive performance, while total vitamin B_12_ concentration has not been shown to be a good predictor, as indicated by a longitudinal cohort study including 1648 senior women and men (≥65 years) over a period of 10 years. An increase of the holo‐TC concentration from 50 to 100 pmol L^−1^ is associated with a 30% lower reduction of cognitive performance; the increase in MMA and homocysteine concentrations from 0.25 to 0.5 µmol L^−1^ and from 10 to 20 µmol L^−1^, respectively, is associated with an impairment of cognitive performance by >50%.^98^

The data from intervention studies on the influence of vitamin B_12_ supplementation on cognitive performance are contradictory; the effects seem to depend on the basal homocysteine concentration and duration of the intervention. A systematic review of 25 randomized controlled studies and cohort studies concluded that there is insufficient evidence regarding an association between vitamin B_12_ intake or vitamin B_12_ status, respectively, and cognitive performance.^94^ Another systematic review of ten observational studies including subjects over 50 years had shown similar results.^99^

## 7 Discussion and Conclusion

### 7.1 Vitamin B_12_ Reference Values—What has Changed?

The reference values for vitamin B_12 _intake from international expert panels vary considerably.^57^, ^100^, ^101^, ^102^ This variance of recommendations results from the fact that the scientific basis allows a certain variation (see **Table** 4). Therefore, they have been the subject of intense controversy. Since the publication of the first D‐A‐CH reference values for the intake of vitamin B_12_, several vitamin B_12_ biomarker studies have questioned whether the current reference value of 3.0 µg vitamin B_12_ d^−1^ is adequate for optimal vitamin B_12_ status in healthy adults.^13^, ^14^, ^15^ From this background, D‐A‐CH nutrition societies have reviewed and evaluated vitamin B_12_‐related biomarker studies and revised the reference values for vitamin B_12_ in 2018.

**Table 4:** Reference values for the intake of vitamin B_12_ from different nutrition societies

	World Health Organisation (WHO), Food and Agricultural Organisation of the United Nations (FAO)^57^	European Food Safety Authority (EFSA)^102^	Institute of Medicine (IoM)^100^	Nordic Council of Ministers^101^
Infants	Infants 0 to 12 months RNI vitamin B_12_ 0 to 6 months: 0.4 µg d^−1^ 7 to 12 months: 0.7 µg d^−1^ Based on the concentrations of vitamin B_12_ in human milk	Infants 0 to under 6 months^127^ AI vitamin B_12_= 0.4 µg d^−1^ Based on the vitamin B_12_ intake in exclusively breast‐fed infants from mothers with adequate B_12_ status Infants 7 to under 11 months AI vitamin B_12_= 1.5 µg d^−1^ Based on upwards extrapolation from the vitamin B_12_ intake in exclusively breast‐fed infants and down‐scaling from the AI for adults	Infants aged 0 to 6 months AI vitamin B_12_= 0.4 µg d^−1^ Based on the vitamin B_12_ intake in exclusively breast‐fed infants from mothers with adequate B_12_ status Infants aged 7 to 12 months AI vitamin B_12_= 0.5 µg d^−1^ Based on upwards extrapolation from the vitamin B_12_ intake in exclusively breast‐fed infants and down‐scaling from the AI for adults	Infants 0 to 23 months RI Vitamin B_12_ < 6 months: ‐ 6 to 11 months: 0.5 µg d^−1^ 12 to 23 months: 0.6 µg d^−1^ No details about derivation and database
Children and adolescents	Children and adolescents 1 to 18 years RNI vitamin B_12_ 1 to 3 years: 0.9 µg d^−1^ 4 to 6 years: 1.2 µg d^−1^ 7 to 9 years: 1.8 µg d^−1^ 10 to 18 years: 2.4 µg d^−1^ Based on the suggestion of the same intakes for adolescents as those for adults with progressive reduction of intake for younger groups	Children and adolescents 1 to 17 years AI vitamin B_12_ 1 to 3 years: 1.5 µg d^−1^ 4 to 6 years: 1.5 µg d^−1^ 7 to 10 years: 2.5 µg d^−1^ 11 to 14 years: 3.5 µg d^−1^ 15 to 17 years: 4 µg d^−1^ Based on downwards extrapolation from the vitamin B_12_‐values for adults	Children and adolescents 1 to 18 years RDA vitamin B_12_ 1 to 3 years: 0.9 µg d^−1^ 4 to 8 years: 1.2 µg d^−1^ 9 to 13 years: 1.8 µg d^−1^ 14 to 18 years: 2.4 µg d^−1^ Based on downwards extrapolation from the vitamin B_12_‐values for adults	Children and adolescents 2 to 17 years RI vitamin B_12_ 2 to 5 years: 0.8 µg d^−1^ 6 to 9 years: 1.3 µg d^−1^ 10 to 13 years: 2.0 µg d^−1^ 14 to 17 years: 2.0 µg d^−1^ Based on 0.05 µg vitamin B_12_ per kg body weight
Adults	Adults ≥ 19 years RNI vitamin B_12_= 2.4 µg d^−1^ Based on the amount of vitamin B_12_ to maintain the body's store	Adults ≥ 18 years AI vitamin B_12_= 4.0 µg d^−1^ Based on an adequate biomarker status of vitamin B_12_ in healthy people	Adults ≥ 19 years RDA vitamin B_12_= 2.4 µg d^−1^ Based on the determination of the amount of vitamin B_12_ needed for maintenance of an adequate erythropoiesis	Adults ≥ 18 years RI vitamin B_12_= 2.0 µg d^−1^ Based on the determination of the amount of vitamin B_12_ needed for maintenance of an adequate erythropoiesis
Pregnant women	RNI vitamin B_12_= 2.6 µg d^−1^ Based on the vitamin B_12_ requirement for the fetus	AI vitamin B_12_= 4.5 µg d^−1^ Based on the vitamin B_12_ requirement for the fetus	RDA vitamin B_12_= 2.6 µg d^−1^ Based on the vitamin B_12_ requirement for the fetus	RI vitamin B_12_= 2.0 µg d^−1^ Based on adequate stores to cover the additional requirement
Lactating women	RNI vitamin B_12_= 2.8 µg d^−1^ Based on the vitamin B_12_ requirement for loss via breast milk	AI vitamin B_12_= 5.0 µg d^−1^ Based on the vitamin B_12_ requirement for loss via breast milk	RDA vitamin B_12_= 2.8 µg d^−1^ Based on the vitamin B_12_ requirement for loss via breast milk	RI vitamin B_12_= 2.6 µg d^−1^ Recommendation to compensate for the loss of vitamin B_12_ in breast milk

AI: Adequate Intake; RDA: Recommended Dietary Allowance; RI: Recommended Intake; RNI: Reference Nutrient Intake.

Developing new reference values for vitamin B_12_ intake requires the definition of the best target biomarkers for a supply, corresponding to adequacy of metabolic functions. For adults, the reference value for vitamin B_12_ intake is based on biomarkers of vitamin B_12_ status, notably adequate serum concentrations of holo‐TC and MMA. As a result, the revised vitamin B_12_ reference value for adults was changed from a recommended intake value of 3 µg d^−1^ up to an estimated value of 4 µg d^−1^, which is in line with the reference value of vitamin B_12_ of the European Food Safety Authority (EFSA).^102^

### 7.2 Vitamin B_12_ Reference Values—Practical Aspects

According to the analysis of the data from the National Nutrition Survey II (NVS II, 2005–2006), the median vitamin B_12_ intake in Germany in women and men between the ages of 15 and 80 years is 3.8 and 5.3 µg d^−1^, respectively.^103^, ^104^

The intake of foods naturally rich in cobalamin as part of a mixed diet can ensure sufficient vitamin B_12_ supply. To achieve sufficient vitamin B_12_ intake, a regular intake of animal‐based food such as milk and dairy products, fish, poultry and lean meat is recommended. It should be noted that during food processing, e.g., cooking or pasteurization, a vitamin B_12_ loss up to 50% might occur.^105^

People adopting a vegan diet that excludes animal foods should be encouraged to take a dietary vitamin B_12_ supplement and should have their vitamin B_12_ status regularly checked by a physician.^106^ Spirulina and other cyanobacteria, which are advertised as natural vitamin B_12_ sources for vegans, do not contain cobalamins that are bioavailable for humans. In addition, the cobalamin analogues that they contain can even inhibit intestinal uptake of vitamin B_12_ and might suppress the formation of biologically active vitamin B_12_ coenzymes.^107^, ^108^ Therefore, they are unsuitable to cover the requirements of vegans.

Partly, vitamin B_12_ deficiency represents a health issue for ovo‐lacto vegetarians.^105^, ^109^, ^110^ Thus, it is recommended that vegetarians should have their vitamin B_12_ status checked regularly by a physician, too, and should take dietary supplements to achieve sufficient vitamin B_12_ supply if necessary.^105^, ^111^, ^112^, ^113^ In particular, vegetarians with increased nutrient requirements, for example, due to pregnancy or lactation, should pay attention to sufficient vitamin B_12_ intake.

In older people, the risk of vitamin B_12_ deficiency is increased, mainly due to impaired absorption because of medication (e.g., proton pump inhibitors, H_2_ blockers) or diseases (e.g., atrophic gastritis) and less due to insufficient dietary intake of vitamin B_12_.^114^, ^115^, ^116^, ^117^ In the case of atrophic gastritis with hypochlorhydria and related malabsorption of dietary protein‐bound vitamin B_12_,^118^, ^119^ supplementation of vitamin B_12_ is recommended to ensure adequate vitamin B_12_ status.^114^, ^116^, ^120^ In contrast to vitamin B_12_ from natural foods, the absorption of crystalline vitamin B_12_ from preparations is not impaired in atrophic gastritis type B‐related hypochlorhydria.^116^, ^120^ However, investigations show that the use of dietary supplements or fortified foods reduces the prevalence of vitamin B_12_ deficiency in older people,^96^, ^121^, ^122^, ^123^ but the risk of insufficient supply in general persists because of the dosage, which is often too low.^124^, ^125^ Thus, a regular observation of the vitamin B_12_ status is also recommended for older people. For the assessment of the vitamin B_12_ status, a relevant functional parameter such as MMA should be combined with a status marker such as serum vitamin B_12_ or holo‐TC.

## Conflict of Interest

Dr. Alexander Ströhle received an honorarium from the German Nutrition Society (DGE) for developing the first draft of the dietary reference values for vitamin B_12_ intake.

## References

[1] Deutsche Gesellschaft für Ernährung, Österreichische Gesellschaft für Ernährung, Schweizerische Gesellschaft für Ernährung (Eds.), Referenzwerte für die Nährstoffzufuh*r*, Bonn 2017.

[2] A. Bechthold , V. Albrecht , E. Leschik‐Bonnet , H. Heseker , Evaluation of vitamin supplies in Germany. Data on vitamin intake, https://www.dge.de/fileadmin/public/doc/ws/statement/130515-DGE-statement-vitamin-supply.pdf (accessed July 18, 2018).

[3] German Nutrition Society , Ann. Nutr. Metab. 2012, 60, 241. 22677925 10.1159/000337547

[4] German Nutrition Society , Ann. Nutr. Metab. 2013, 63, 186.24356454

[5] M. B. Krawinkel , D. Strohm , A. Weissenborn , B. Watzl , M. Eichholzer , K. Bärlocher , I. Elmadfa , E. Leschik‐Bonnet , H. Heseker , Eur. J. Clin. Nutr. 2014, 68, 719. 24690591 10.1038/ejcn.2014.45 PMC4050524

[6] German Nutrition Society , Ann. Nutr. Metab. 2015, 67, 13. 26414007 10.1159/000437243

[7] German Nutrition Society , Ann. Nutr. Metab. 2015, 66, 219. 26414007 10.1159/000437243

[8] A. P. Kipp , D. Strohm , R. Brigelius‐Flohe , Schomburg, L., A. Bechthold , E. Leschik‐Bonnet , H. Heseker , J. Trace Elem. Med. Biol. 2015, 32, 195. 26302929 10.1016/j.jtemb.2015.07.005

[9] D. Strohm , A. Bechthold , N. Isik , E. Leschik‐Bonnet , H. Heseker , NFS Journal 2016, 3, 20.

[10] D. Strohm , A. Bechthold , S. Ellinger , E. Leschik‐Bonnet , P. Stehle , H. Heseker , German Nutrition Society (DGE) , Ann. Nutr. Metab. 2018, 72, 12. 29232668 10.1159/000484355 PMC6008876

[11] D. Strohm , S. Ellinger , E. Leschik‐Bonnet , F. Maretzke , H. Heseker , Ann. Nutr. Metab. 2017, 71, 118. 28803230 10.1159/000479705 PMC5639605

[12] Deutsche Gesellschaft für Ernährung, ÖGE, SGE, SVE (Eds.), *Referenzwerte für die Nährstoffzufuhr* , Umschau Verlag, Frankfurt/M. 2000.

[13] M. V. Bor , Kvon. Castel‐Roberts , G. P. Kauwell , S. P. Stabler , R. H. Allen , D. R. Maneval , L. B. Bailey , E. Nexo , Am. J. Clin. Nutr. 2010, 91, 571. 20071646 10.3945/ajcn.2009.28082

[14] K. Pentieva , C. Hughes , N. Askin , L. Hoey , A. Molloy , J. Scott , H. McNulty , Proc Nutr Soc 2012, 71, E318.

[15] M. V. Bor , E. Lydeking‐Olsen , J. Møller , E. Nexø , Am. J. Clin. Nutr. 2006, 83, 52. 16400049 10.1093/ajcn/83.1.52

[16] L. Randaccio , S. Geremia , N. Demitri , J. Wuerges , Molecules 2010, 15, 3228. 20657474 10.3390/molecules15053228 PMC6257451

[17] F. Watanabe , Exp. Biol. Med. 2007, 232, 1266. 10.3181/0703-MR-67 17959839

[18] I. Chanarin , The megaloblastic anaemias, Blackwell Scientific, Oxford 1979.

[19] R. Obeid , S. N. Fedosov , E. Nexo , Mol. Nutr. Food Res. 2015, 59, 1364. 25820384 10.1002/mnfr.201500019 PMC4692085

[20] M. V. Bor , E. Nexo , In: Herrmann W., Obeid R. (Eds.), Vitamins in the prevention of human diseases, De Gruyter, Berlin 2011, 187.

[21] W. Herrmann , R. Obeid , In: Herrmann W., Obeid R. (Eds.), Vitamins in the prevention of human diseases, De Gruyter, Berlin 2011, 213.

[22] R. Carmel , In: Ross A. C., Caballero B., Cousins R. J., Tucker K. L., Ziegler T. R. (Eds.), Modern nutrition in health and disease, Lippincott Williams & Wilkins, Philadelphia 2014, 369.

[23] P. Reizenstein , G. Ek , C. M. Matthews , Phys. Med. Biol. 1966, 11, 295. 5954616 10.1088/0031-9155/11/2/309

[24] E. V. Quadros , Br. J. Haematol. 2010, 148, 195. 19832808 10.1111/j.1365-2141.2009.07937.x PMC2809139

[25] E. V. Quadros , J. M. Sequeira , Biochimie 2013, 95, 1008‐1018. 23415653 10.1016/j.biochi.2013.02.004 PMC3902480

[26] V. Devalia , M. S. Hamilton , A. M. Molloy , Br. J. Haematol. 2014, 166, 496. 24942828 10.1111/bjh.12959

[27] R. Carmel , Am. J. Clin. Nutr. 2011, 94, 348S. 21593511 10.3945/ajcn.111.013441 PMC3174853

[28] Y. Lamers , Curr. Opin. Clin. Nutr. Metab. Care 2011, 14, 445. 21832901 10.1097/MCO.0b013e328349f9a7

[29] R. Green , Am. J. Clin. Nutr. 2011, 94, (Suppl), 666S. 21733877 10.3945/ajcn.110.009613 PMC3142735

[30] E. A. Yetley , C. M. Pfeiffer , K. W. Phinney , Z. Fazili , D. A. Lacher , R. L. Bailey , S. Blackmore , J. L. Bock , L. C. Brody , R. Carmel , L. R. Curtin , R. A. Durazo‐Arvizu , J. H. Eckfeldt , R. Green , J. F. Gregory III , A. N. Hoofnagle , D. W. Jacobsen , P. F. Jacques , A. M. Molloy , J. Massaro , J. L. Mills , E. Nexo , J. I. Rader , J. Selhub , C. Sempos , B. Shane , S. Stabler , P. Stover , T. Tamura , A. Tedstone , S. J. Thorpe , P. M. Coates , C. L. Johnson , M. F. Picciano , Am. J. Clin. Nutr. 2011, 94, (Suppl), 303S. 21593502 10.3945/ajcn.111.013011 PMC3127517

[31] W. Herrmann , R. Obeid , Deutsches Ärzteblatt international 2008, 105, 680. 19623286 10.3238/arztebl.2008.0680 PMC2696961

[32] W. Herrmann , R. Obeid , Eur. J. Clin. Invest. 2013, 43, 231. 23330849 10.1111/eci.12034

[33] R. Aparicio‐Ugarriza , G. Palacios , M. Alder , M. González‐Gross , Clinical Chemistry and Laboratory Medicine (CCLM) 2015, 53, 1149. 25470607 10.1515/cclm-2014-0784

[34] L. Hannibal , V. Lysne , A.‐L. Bjorke‐Monsen , S. Behringer , S. C. Grunert , U. Spiekerkoetter , D. W. Jacobsen , H. J. Blom , Front. Mol. Biosci. 2016, 3, 27. 27446930 10.3389/fmolb.2016.00027 PMC4921487

[35] R. Carmel , Biochimie 2013, 95, 1047‐1055. 23416723 10.1016/j.biochi.2013.02.008

[36] R. Green , L. H. Allen , A.‐L. Bjørke‐Monsen , A. Brito , J.‐L. Guéant , J. W. Miller , A. M. Molloy ; E. Nexo , S. Stabler , B.‐H. Toh , P. M. Ueland ; C. Yajnik , V. B. deficiency . Nature Reviews Disease Primers 2017, 3, 17040. 10.1038/nrdp.2017.40 28660890

[37] R. L. Bailey , R. A. Durazo‐Arvizu , R. Carmel , R. Green , C. M. Pfeiffer , C. T. Sempos , A. Carriquiry , E. A. Yetley , Am. J. Clin. Nutr. 2013, 98, 460. 23803883 10.3945/ajcn.113.061234 PMC3712554

[38] E. V. Quadros , Y. Nakayama , J. M. Sequeira , Blood 2009, 113, 186. 18779389 10.1182/blood-2008-05-158949 PMC2614632

[39] E. Nexo , E. Hoffmann‐Lucke , Am. J. Clin. Nutr. 2011, 94, 359S. 21593496 10.3945/ajcn.111.013458 PMC3127504

[40] S. N. Fedosov , A. Brito , J. W. Miller , R. Green , L. H. Allen , Clinical Chemistry and Laboratory Medicine (CCLM) 2015, 53, 1215. 25720072 10.1515/cclm-2014-0818

[41] R. Green , L. J. Kinsella , Neurology 1995, 45, 1435. 7644036 10.1212/wnl.45.8.1435

[42] J. B. Ubbink , W. J. Vermaak , A. van der Merwe , P. J. Becker , Am. J. Clin. Nutr. 1993, 57, 47. 8416664 10.1093/ajcn/57.1.47

[43] B. de Benoist , Food Nutr. Bull. 2008, 29, S238. 18709899 10.1177/15648265080292S129

[44] E. Bächli , J. Fehr , Schweiz Med Wochenschr 1999, 129, 861. 10420442

[45] O. Stanger , W. Herrmann , K. Pietrzik , B. Fowler , J. Geisel , J. Dierkes , M. Weger , Clin. Chem. Lab. Med. 2003, 41, 1392. 14656016 10.1515/CCLM.2003.214

[46] E. L. Doets , P. H. In ‚t Veld , H. Paulette , A. Szczecińska , R. A. M. Dhonukshe‐Rutten , A. E. J. M. Cavelaars , P. van ’t Veer , A. Brzozowska , L. C. P. G.M. de Groot , Ann. Nutr. Metab. 2013, 62, 311. 23796635 10.1159/000346968

[47] L. H. Allen , Int. J. Vitam. Nutr. Res. 2010, 80, 330. 21462117 10.1024/0300-9831/a000041

[48] R. H. Allen , S. P. Stabler , Am. J. Clin. Nutr. 2008, 87, 1324. 18469256 10.1093/ajcn/87.5.1324 PMC2900183

[49] M. Wolters , S. Hermann , A. Hahn , Am. J. Clin. Nutr. 2003, 78, 765. 14522735 10.1093/ajcn/78.4.765

[50] WHO (World Health Organization) , *Protein and amino acid requirements in human nutrition: Report of a joint WHO/FAO/UNU expert consultation* , Genf 2007. 18330140

[51] C. Bührer , O. Genzel‐Boroviczény , F. Jochum , T. Kauth , M. Kersting , B. Koletzko , W. Mihatsch , H. Przyrembel , T. Reinehr , P. Zimmer , Monatsschr. Kinderheilkd. 2014, 162, 527.

[52] N. F. Butte , M. G. Lopez‐Alarcon , C. Garza , *Nutrient adequacy of exclusive breastfeeding for the term infant during the first six months of life* , www.who.int/nutrition/publications/infantfeeding/nut_adequacy_of_exc_bfeeding_eng.pdf (accessed July 18, 2018).

[53] E. Greibe , D. L. Lildballe , S. Streym , P. Vestergaard , L. Rejnmark , L. Mosekilde , E. Nexo , Am. J. Clin. Nutr. 2013, 98, 389. 23783295 10.3945/ajcn.113.058479

[54] K. L. Deegan , K. M. Jones , C. Zuleta , M. Ramirez‐Zea , D. L. Lildballe , E. Nexo , L. H. Allen , J. Nutr. 2012, 142, 112. 22131550 10.3945/jn.111.143917

[55] A.‐L. Bjørke‐Monsen , P. M. Ueland , J. Inherit. Metab. Dis. 2011, 34, 111. 20508991 10.1007/s10545-010-9119-1

[56] M. C. Neville , R. Keller , J. Seacat , V. Lutes , M. Neifert , C. Casey , J. Allen , P. Archer , Am. J. Clin. Nutr. 1988, 48, 1375. 3202087 10.1093/ajcn/48.6.1375

[57] WHO (World Health Organization) , FAO (Food and Agriculture Organization) (Eds.), Vitamin and mineral requirements in human nutrition, Bangkok 2004.

[58] Y. He , Y. Li , Y. Chen , L. Feng , Z. Nie , Nutr. Metab. Cardiovasc. Dis. 2014, 24, 1158. 24984821 10.1016/j.numecd.2014.05.011

[59] Homocysteine Studies Collaboration , JAMA 2002, 288, 2015. 12387654 10.1001/jama.288.16.2015

[60] L. L. Humphrey , R. Fu , K. Rogers , M. Freeman , M. Helfand , Mayo Clin. Proc. 2008, 83, 1203. 18990318 10.4065/83.11.1203

[61] M. Grassi , D. Assanelli , A. Pezzini , Thromb. Res. 2007, 120, 61. 16908053 10.1016/j.thromres.2006.06.016

[62] X. Hou , X. Chen , J. Shi , Gene 2015, 565, 39. 25839940 10.1016/j.gene.2015.03.062

[63] D. S. Wald , J. P. Bestwick , N. J. Wald , Clin. Chem. 2012, 58, 1488. 22896715 10.1373/clinchem.2012.191791

[64] D. S. Wald , J. K. Morris , N. J. Wald , PLoS One 2011, 6, e16473. 21311765 10.1371/journal.pone.0016473 PMC3032783

[65] B. Debreceni , L. Debreceni , Cardiovasc. Ther. 2014, 32, 130. 24571382 10.1111/1755-5922.12064

[66] T. Huang , Y. Chen , B. Yang , J. Yang , M. L. Wahlqvist , D. Li , Clin Nutr 2012, 31, 448. 22652362 10.1016/j.clnu.2011.01.003

[67] Y. Ji , S. Tan , Y. Xu , A. Chandra , C. Shi , B. Song , J. Qin , Y. Gao , Neurology 2013, 81, 1298‐1307. 24049135 10.1212/WNL.0b013e3182a823cc

[68] A. J. Martí‐Carvajal , I. Solà , D. Lathyris , The Cochrane Database of Systematic Reviews 2015, 1, CD006612. 25590290 10.1002/14651858.CD006612.pub4

[69] M. Fenech , Mutation Research/Fundamental and Molecular Mechanisms of Mutagenesis 2012, 733, 21.22093367

[70] W. Wu , S. Kang , D. Zhang , Br. J. Cancer 2013, 109, 1926. 23907430 10.1038/bjc.2013.438 PMC3790153

[71] S. Uccella , A. Mariani , A. H. Wang , R. A. Vierkant , K. Robien , K. E. Anderson , J. R. Cerhan , Ann. Oncol. 2011, 22, 2129. 21324952 10.1093/annonc/mdq724 PMC3202166

[72] J. J. Liu , A. Hazra , E. Giovannucci , S. E. Hankinson , B. Rosner , Ide. Vivo , Br. J. Cancer 2013, 108, 183. 23299529 10.1038/bjc.2012.534 PMC3553527

[73] A. A. Razzak , A. S. Oxentenko , R. A. Vierkant , L. S. Tillmans , A. H. Wang , D. J. Weisenberger , P. W. Laird , C. F. Lynch , K. E. Anderson , A. J. French , R. W. Haile , L. J. Harnack , J. D. Potter , S. L. Slager , T. C. Smyrk , S. N. Thibodeau , J. R. Cerhan , P. J. Limburg , Nutr. Cancer 2012, 64, 899. 23061900 10.1080/01635581.2012.714833 PMC3584680

[74] T. J. Key , P. N. Appleby , G. Masset , E. J. Brunner , J. E. Cade , D. C. Greenwood , A. M. Stephen , D. Kuh ; A. Bhaniani , N. Powell , K.‐T. Khaw , Int. J. Cancer 2012, 131, E320. 22139959 10.1002/ijc.27386

[75] L. Le Marchand , K. K. White , A. M. Y. Nomura , L. R. Wilkens , J. S. Selhub , M. Tiirikainen , M. T. Goodman , S. P. Murphy , B. E. Henderson , L. N. Kolonel , Cancer Epidemiol., Biomarkers Prev. 2009, 18, 2195. 19661077 10.1158/1055-9965.EPI-09-0141 PMC2978659

[76] A. M. Dahlin , B. van Guelpen , J. Hultdin , I. Johansson , G. Hallmans , R. Palmqvist , Int. J. Cancer 2008, 122, 2057. 18092327 10.1002/ijc.23299

[77] S. J. Weinstein , D. Albanes , J. Selhub , B. Graubard , U. Lim , P. R. Taylor , J. Virtamo , R. Stolzenberg‐Solomon , Cancer Epidemiol., Biomarkers Prev. 2008, 17, 3233. 18990766 10.1158/1055-9965.EPI-08-0459 PMC2656360

[78] B. Mao , Y. Li , Z. Zhang , C. Chen , Y. Chen , C. Ding , L. Lei , J. Li , M. Jiang , D. Wang , G. Wang , PLoS One 2015, 10, e0141762. 26513161 10.1371/journal.pone.0141762 PMC4625965

[79] S. M. Collin , C. Metcalfe , H. Refsum , S. J. Lewis , L. Zuccolo , G. D. Smith , L. Chen , R. Harris , M. Davis , G. Marsden , C. Johnston , J. A. Lane , M. Ebbing , K. H. Bonaa , O. Nygard , P. M. Ueland , M. V. Grau , J. A. Baron , J. L. Donovan , D. E. Neal , F. C. Hamdy , A. D. Smith , R. M. Martin , Cancer Epidemiol., Biomarkers Prev. 2010, 19, 1632. 20501771 10.1158/1055-9965.EPI-10-0180 PMC3759018

[80] J. F. Arendt , L. Pedersen , E. Nexo , H. T. Sørensen , JNCI: Journal of the National Cancer Institute 2013, 105, 1799. 24249744 10.1093/jnci/djt315 PMC3848986

[81] R. Obeid , K. Pietrzik , Re: A. J. Price , R. C. Travis , P. N. Appleby , et al. 10.1016/j.eururo.2016.03.029: Serum concentrations of folate and vitamin B_12_ and the risk of prostate cancer according to pooled data: The devil Is in the detail. Eur. Urol. 2016, 70, e133. 27236495

[82] M. Puri , L. Kaur , G. K. Walia , R. Mukhopadhhyay , M. P. Sachdeva , S. S. Trivedi , P. K. Ghosh , K. N. Saraswathy , J. Perinat. Med. 2013, 41, 549. 23612630 10.1515/jpm-2012-0252

[83] U. Hübner , A. Alwan , M. Jouma , M. Tabbaa , H. Schorr , W. Herrmann , Clin. Chem. Lab. Med. 2008, 46, 1265–1269. 18636794 10.1515/CCLM.2008.247

[84] Z.‐P. Wang , X.‐X. Shang , Z.‐T. Zhao , J. Maternal‐Fetal Neonat. Med. 2012, 25, 389. 10.3109/14767058.2011.580800 21627554

[85] K.‐F. Tang , Y.‐L. Li , H.‐Y. Wang , Sci. Rep. 2015, 5, 8510. 25728980 10.1038/srep08510 PMC4345334

[86] J. L. Finkelstein , A. J. Layden , P. J. Stover , Adv. Nutrition 2015, 6, 552. 10.3945/an.115.008201 PMC4561829 26374177

[87] T. P. Vacek , A. Kalani , M. J. Voor , S. C. Tyagi , N. Tyagi , Clin. Chem. Lab. Med. 2013, 51, 579. 23449525 10.1515/cclm-2012-0605 PMC3951268

[88] Z. Dai , W.‐P. Koh , Nutrients 2015, 7, 3322‐3346. 25961321 10.3390/nu7053322 PMC4446754

[89] M. Herrmann , J. Schmidt , N. Umanskaya , G. Colaianni , F. Al Marrawi , T. Widmann , A. Zallone , B. Wildemann , W. Herrmann , Bone 2007, 41, 584. 17681876 10.1016/j.bone.2007.06.005

[90] S. Ozdem , S. Samanci , A. Tasatargil , A. Yildiz , G. Sadan , L. Donmez , M. Herrmann , Scand. J. Clin. Lab. Invest. 2007, 67, 748. 17852810 10.1080/00365510701342088

[91] J. P. van Wijngaarden , E. L. Doets , A. Szczecińska , O. W. Souverein , M. E. Duffy , C. Dullemeijer , A. E. J. M. Cavelaars , B. Pietruszka , P. van't Veer , A. Brzozowska , R. A. M. Dhonukshe‐Rutten , C. P. G.Mde. Groot , J. Nutr. Metab. 2013, 2013, 1. 10.1155/2013/486186 PMC3590816 23509616

[92] J. Ruan , X. Gong , J. Kong , H. Wang , X. Zheng , T. Chen , Med. Sci. Monit. 2015, 21, 3048. 25805360 10.12659/MSM.893310 PMC4384513

[93] F. O‘Leary , M. Allman‐Farinelli , S. Samman , Br. J. Nutr. 2012, 108, 1948. 23084026 10.1017/S0007114512004175

[94] E. L. Doets , J. P. van Wijngaarden , A. Szczecińska , C. Dullemeijer , O. W. Souverein , R. A. M. Dhonukshe‐Rutten , A. E. J. M. Cavelaars , P. van ’t Veer , A. Brzozowska , L. C. P. G.M. de Groot , Epidemiol. Rev. 2013, 35, 2. 23221971 10.1093/epirev/mxs003

[95] A. Vogiatzoglou , H. Refsum , C. Johnston , S. M. Smith , K. M. Bradley , Cde. Jager , M. M. Budge , A. D. Smith , Neurology 2008, 71, 826. 18779510 10.1212/01.wnl.0000325581.26991.f2

[96] A. Vogiatzoglou , A. D. Smith , E. Nurk , P. Berstad , C. A. Drevon , P. M. Ueland , S. E. Vollset , G. S. Tell , H. Refsum , Am. J. Clin. Nutr. 2009, 89, 1078. 19190073 10.3945/ajcn.2008.26598

[97] A. D. Smith , S. M. Smith , C.Ade. Jager , P. Whitbread , C. Johnston , G. Agacinski , A. Oulhaj , K. M. Bradley , R. Jacoby , H. Refsum , PLoS One 2010, 5, e12244. 20838622 10.1371/journal.pone.0012244 PMC2935890

[98] R. Clarke , J. Birks , E. Nexo , P. M. Ueland , J. Schneede , J. Scott , A. Molloy , J. G. Evans , Am. J. Clin. Nutr. 2007, 86, 1384. 17991650 10.1093/ajcn/86.5.1384

[99] L. M. Miles , K. Mills , R. Clarke , A. D. Dangour , Br. J. Nutr. 2015, 114, 503. 26202329 10.1017/S0007114515002226

[100] Dietary Reference Intakes for Thiamin, Riboflavin, Niacin, Vitamin B6, Folate, Vitamin B12, Pantothenic acid, Biotin, and Choline (Ed: IOM (Institute of Medicine ), National Academies Press, Washington, DC 1998. 23193625

[101] Nordic Council of Ministers (Ed.), *Nordic Nutrition Recommendations 2012. Integrating nutrition and physical activity* , Kopenhagen 2014.

[102] EFSA (European Food Safety Authority) , EFSA Journal 2015, 13, 4150. 10.2903/j.efsa.2015.4139 PMC7163586 32313571

[103] C. Krems , C. Walter , T. Heuer , I. Hoffmann , In: Deutsche Gesellschaft für Ernährung (Ed.), *12. Ernährungsbericht 2012* , Bonn 2012, pp. 40.

[104] MRI (Max Rubner‐Institut , Bundesforschungsinstitut für Ernährung und Lebensmittel), Energie‐ und Nährstoffzufuhr bei Jugendlichen und Erwachsenen in der NVS II. Email 2014, Karlsruhe 2014.

[105] G. Rizzo , A. S. Lagana , A. M. C. Rapisarda , G. M. G. La Ferrera , M. Buscema , P. Rossetti , A. Nigro , V. Muscia , G. Valenti , F. Sapia , G. Sarpietro , M. Zigarelli , S. G. Vitale , Nutrients, 2016, 8, 767. 10.3390/nu8120767 PMC5188422 27916823

[106] M. Richter , H. Boeing , D. Grünewald‐Funk , H. Heseker , A. Kroke , E. Leschik‐Bonnet , H. Oberritter , D. Strohm , B. Watzl , Ernahrungs Umschau 2016, 63, 92.

[107] V. Herbert , Am. J. Clin. Nutr. 1988, 48, 852. 3046314 10.1093/ajcn/48.3.852

[108] F. Watanabe , Y. Yabuta , Y. Tanioka , T. Bito , J. Agric. Food Chem. 2013, 61, 6769. 23782218 10.1021/jf401545z

[109] A. M. J. Gilsing , F. L. Crowe , Z. Lloyd‐Wright , T. A. B. Sanders , P. N. Appleby , N. E. Allen , T. J. Key , Eur. J. Clin. Nutr. 2010, 64, 933. 20648045 10.1038/ejcn.2010.142 PMC2933506

[110] W. Herrmann , H. Schorr , R. Obeid , J. Geisel , Am. J. Clin. Nutr. 2003, 78, 131. 12816782 10.1093/ajcn/78.1.131

[111] A. Ströhle , C. Löser , I. Behrendt , C. Leitzmann , A. Hahn , Aktuelle Ernährungsmedizin 2016, 41, 47.

[112] R. Pawlak , S. J. Parrott , S. Raj , D. Cullum‐Dugan , D. Lucus , Nutr. Rev. 2013, 71, 110. 23356638 10.1111/nure.12001

[113] A. Ströhle , A. Hahn , Med Monatschr Pharm 2018, 41, 113.

[114] P. J. Stover , Curr. Opin. Clin. Nutr. Metab. Care 2010, 13, 24. 19904199 10.1097/MCO.0b013e328333d157 PMC5130103

[115] C. F. Hughes , M. Ward , L. Hoey , H. McNulty , Annals of Clin. Biochem.: Int. J. Biochem. Lab. Med. 2013, 50, 315. 10.1177/0004563212473279 23592803

[116] M. Wolters , A. Ströhle , A. Hahn , Prev. Med. 2004, 39, 1256. 15539065 10.1016/j.ypmed.2004.04.047

[117] R. Conzade , W. Koenig , M. Heier , A. Schneider , E. Grill , A. Peters , B. Thorand , Nutrients 2017, 9, 1276. 10.3390/nu9121276 PMC5748727 29168737

[118] A. Hurwitz , D. A. Brady , S. E. Schaal , I. M. Samloff , J. Dedon , C. E. Ruhl , JAMA 1997, 278, 659. 9272898

[119] S. D. Krasinski , R. M. Russell , I. M. Samloff , R. A. Jacob , G. E. Dallal , R. B. McGandy , S. C. Hartz , J. Am. Geriatr. Soc. 1986, 34, 800. 3771980 10.1111/j.1532-5415.1986.tb03985.x

[120] H. W. Baik , R. M. Russell , Annu. Rev. Nutr. 1999, 19, 357. 10448529 10.1146/annurev.nutr.19.1.357

[121] A. Garcia , A. Paris‐Pombo , L. Evans , A. Day , M. Freedman , J. Am. Geriatr. Soc. 2002, 50, 1401. 12164997 10.1046/j.1532-5415.2002.50362.x

[122] S. Rajan , J. I. Wallace , S. A. Beresford , K. I. Brodkin , R. A. Allen , S. P. Stabler , J. Am. Geriatr. Soc. 2002, 50, 624‐630. 11982661 10.1046/j.1532-5415.2002.50155.x

[123] L. L. Kwan , O. I. Bermudez , K. L. Tucker , J. Nutr. 2002, 132, 2059. 12097693 10.1093/jn/132.7.2059

[124] E. C. Seal , J. Metz , L. Flicker , J. Melny , J. Am. Geriatr. Soc. 2002, 50, 146. 12028259 10.1046/j.1532-5415.2002.50020.x

[125] S. Rajan , J. I. Wallace , K. I. Brodkin , S. A. Beresford , R. H. Allen , S. P. Stabler , J. Am. Geriatr. Soc. 2002, 50, 1789. 12410896 10.1046/j.1532-5415.2002.50506.x

[126] RKI (Robert Koch‐Institut) , Referenzperzentile für anthropometrische Maßzahlen und Blutdruck aus der Studie zur Gesundheit von Kindern und Jugendlichen in Deutschland (KiGGS) 2003–2006, Berlin 2011.

[127] EFSA (European Food Safety Authority) , EFSA Journal 2013, 11, 3408.

